# Yamane Technique Versus Prolene Mesh for Intraocular Lens Scleral Fixation in Aphakia

**DOI:** 10.1155/joph/3731656

**Published:** 2026-02-17

**Authors:** Islam Awny, Elshimaa A. Mateen Mossa, Islam Saad EL Saman, Alaa Abdalsadek Ahmed Sinjab, Amr Mounir

**Affiliations:** ^1^ Ophthalmology Department, Sohag Faculty of Medicine, Sohag University, Sohag, Egypt, sohag-univ.edu.eg

**Keywords:** aphakia, prolene mesh, ultrasound biomicroscopy, Yamane technique

## Abstract

**Purpose:**

To evaluate transscleral prolene mesh in aphakic patients lacking capsular support by comparing this technique with the Yamane technique.

**Methods:**

A comparative, prospective, randomized study was conducted on 40 eyes of 40 patients who were aphakic without capsular support. Aphakia is either posttraumatic or after complicated cataract surgery with partial or total loss of capsular support. Patients were randomly assigned to one of two groups based on the surgical intervention they received: Group A (Yamane technique) and Group B (transscleral prolene mesh technique). All patients were followed up for 3 months after surgery. Postoperative visual acuity, refraction, IOL centration, and complications were documented during the follow‐up period.

**Results:**

Patients in both groups were comparable for both ages, 55.78 (± 8.63) for Group A and 59.10 (± 9.95) for Group B, and gender. In Group A, 50% were males and 50% were females. In Group B, 60% were males and 40% were females. After excluding complicated cases, both groups were compared for preoperative and postoperative results (UCVA, BCVA, IOP, refractive errors, and IOL tilt). The two groups had no statistically significant differences (*p* value ≥ 0.05). Moreover, refractive errors (sphere and cylinder) showed a strong negative correlation to IOL tilt of more than 5° for both groups. Seven patients out of twenty were complicated in Group A. Three patients had IOL tilt > 5°, 2 had IOL decentration > 0.2 mm, and two had pseudophakic reverse pupillary block. Four patients out of twenty had complications in Group B. Two patients had IOL tilt > 5°, and the rest had IOL decentration > 0.2 mm.

**Conclusion:**

The prolene mesh technique showed a considerable improvement in visual outcome compared to the Yamane technique and may even offer a safer option with fewer predictable complications.

**Trial Registration:** ClinicalTrials.gov idendifier: NCT06363448

## 1. Introduction

Implanting an intraocular lens (IOL) in the absence of adequate capsular or zonular support remains one of the most challenging situations in cataract surgery. Several surgical options have been developed to address inadequate capsular support, including anterior chamber lens (ACIOLs), iris‐fixated IOLs, and scleral fixation techniques [1, 2].

However, posterior chamber IOL implantation is generally preferred because it maintains a more physiological anatomic position, preserves normal pupillary function, minimizes optical aberrations, reduces the risk of postoperative glaucoma, and provides a safer long‐term environment for patients with compromised corneas [3, 4].

Among posterior chamber fixation techniques, the double‐needle, sutureless intrascleral haptic fixation (ISHF) technique was first described by Yamane et al. in 2017 and has gained wide popularity [5]. It offers a small incision, reduced surgery time, and avoidance of sutures. Nevertheless, despite these advantages, the technique relies on a two‐point fixation construct, which may predispose to IOL decentration, tilt, pupillary capture, and associated complications such as pigment dispersion syndrome, uveitis–glaucoma–hyphema (UGH) syndrome, cystoid macular edema, and undesirable refractive outcomes. These limitations highlight the need for more stable posterior chamber fixation methods, particularly in eyes lacking capsular support [6].

An alternative scleral fixation approach is the transscleral double rectangular suture sulcus reconstruction (DRSSR), which creates a supportive suture‐based mesh 1 mm posterior to the limbus to cradle the IOL optic within the sulcus. This technique theoretically offers improved stability of the IOL by providing a broader support platform and reducing the likelihood of tilt or decentration. Although DRSSR has been described previously, comprehensive comparative studies evaluating its clinical outcomes relative to the Yamane technique remain limited [7, 8].

Given these gaps, there is a need to directly compare the two techniques to determine whether the broader support provided by DRSSR translates into better IOL stability and fewer postoperative complications than the two‐point Yamane fixation.

Therefore, the aim of this study is to compare both techniques in aphakic eyes lacking capsular support with respect to best‐corrected visual acuity (BCVA), postoperative intraocular pressure (IOP), refractive outcomes, intra‐ and postoperative complications, and postoperative IOL position using ultrasound biomicroscopy (UBM).

## 2. Patients and Methods

This is a comparative, prospective, randomized study conducted on 40 eyes of 40 patients who were aphakic without capsular support.

The study was conducted from January 2024 to June 2024. Written consent from the participants was obtained after discussing the ophthalmological examination and the surgical procedure planned to be conducted. Sohag University Local Ethics Committee approved the study protocol (Soh‐Med‐24‐3‐04PD). The study was conducted in accordance with the ethical principles of the Declaration of Helsinki.

This study included patients who were aphakic with insufficient capsular or zonular support, making conventional in the bag IOL implantation impossible. Eligible patients were adults above 40 years of age who developed aphakia either following complicated cataract surgery with partial or total capsular loss or as a consequence of ocular trauma. Only patients with clear ocular media that allowed safe surgical manipulation and accurate postoperative assessment were enrolled.

Included eyes were required to have intact retina, with the ability to undergo complete ophthalmic examination, optical biometry, and postoperative UBM imaging. Patients were recruited from the ophthalmology clinics of Sohag University Hospitals, and only those willing to provide written informed consent were included in the study.

Patients were excluded if they had undergone any previous intraocular surgery other than cataract extraction or if they presented with uncontrolled glaucoma, compromised corneal endothelial status on specular microscopy, retinal or choroidal detachment, retained intraocular foreign body, active or past uveitis, scleromalacia, rubeosis iridis, or any systemic disorder that could negatively impact visual outcomes.

The cases were randomly assigned to one of two groups based on the surgical intervention they received: Group A (Yamane technique) [9] and Group B (transscleral prolene mesh technique).

All surgeries were performed by IA and AS, experienced ophthalmic surgeons, under standard aseptic conditions. Postoperative management included topical antibiotics, corticosteroids, and routine follow‐up assessments.

All cases were assessed and examined ophthalmologically for uncorrected visual acuity and BCVA using Snellen’s chart, the measurement of IOP using Goldmann applanation tonometry, refraction (sphere and cylinder), slit‐lamp examination for the assessment of the anterior segment (cornea, iris, and capsule status), and detailed fundus examination including retinal periphery by indirect ophthalmoscope by expert retinal surgeon.

All patients had preoperative optical biometry for IOL calculation: axial length (AL), keratometry values (K1 and K2), ACD, and IOL power were obtained with Zeiss “IOL MASTER 700.”

One month postoperatively, patients underwent UBM using Sonomed 35‐MHz high‐frequency ultrasound system (Sonomed, Inc., New York, USA). They were positioned supine and received topical anesthetic with benoxinate hydrochloride eye drops. The operation was carried out by a skilled specialist situated on the patient’s right side. The patient’s head was gently steadied by an assistant, and the eyes were studied by an ultrasonic probe equipped with a standard plastic shell.

### 2.1. Surgical Technique

In the second technique (Group B), 360‐degree conjunctival peritomy was done, creating a striding limbus. For the right eye, we started with the nasal and temporal horizontal meridian. The needle of a 10–0 polypropylene (prolene) suture with straight 16.0 mm STC‐6 needles (Ethicon Inc., Ethicon, CA, USA) was introduced transscleral in partial‐thickness sclera from Point A, 2.0 mm from limbus superior‐nasally to exit inferior‐nasally at Point B, which was situated 4 mm inferior to A. The needle was then reintroduced from Point B 2.0 mm posterior to the limbus to the pupillary plane. After that, it was pulled out of the eye by being engaged in the barrel of a 27‐G needle introduced from the opposite exit at Point C. The needle was grasped with a needle holder and reintroduced from the same point for 4 mm in partial‐thickness sclera superiorly to exit 2.0 mm posterior to the superior‐temporal limbus in the superior quadrant of the eye at Point D.

The second needle was reintroduced from the same point of sclera entry, superior‐temporally, in a similar manner, 2.0 mm posterior to the limbus from the superior‐temporal quadrant to the superior‐nasal quadrant across the pupillary area to exit at the same Point A of the previous suture, where a triple throw knot was fashioned and secured with a stay suture. At this same point of exit, a radial sclera tunnel fashioned previously was used to bury the knot once secured.

The same procedure was repeated between the superior and inferior vertical meridians of the eyeball. We started at superior‐nasal entry Point E, 2.0 mm posterior to the limbus via the pupillary plane, where the needle exited 2.0 mm behind the inferior‐nasal limbus at Point F, then passed through partial‐thickness sclera to the inferotemporal limbus at Point G, 4 mm distant from F. The second needle would be passed from the inferior‐temporal Point G and reintroduced through the pupillary plane to the opposite superior‐temporal quadrant at Point H, 2.0 mm behind the limbus. From Point H, the needle was then passed transscleral to Point E, which is 4 mm distant from H. A knot was fashioned and buried in the partial‐thickness scleral groove as mentioned previously.

**FIGURE 1:**
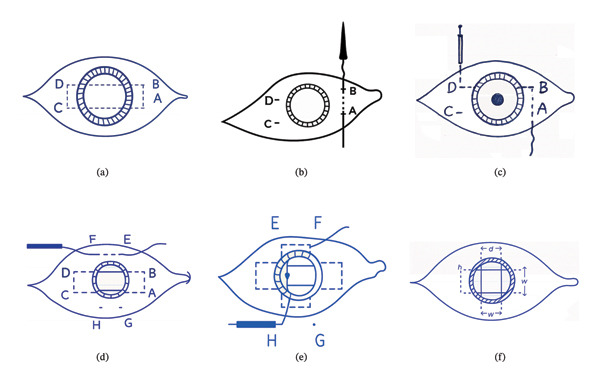
Step‐by‐step illustration of the Prolene mesh technique for scleral fixation of intraocular lens, including mesh preparation and scleral anchoring.

The result of the suturing technique was the creation of a 4 mm square 2^∗^2 prolene meshwork behind the pupillary plane, providing adequate IOL‐optic support, in addition to the peripheral support for the haptics. Foldable three‐piece IOLs (Tecnis ZA9003; Abbott, Illinois, USA) with an optic diameter of 6 mm and an overall diameter of 13 mm were used (Figure 1) (a video file for the transscleral prolene mesh technique is uploaded as a supporting file, “Video 1”).

A subconjunctival injection of short‐acting steroids combined with atropine was routinely administered, followed by patching the eye for a day. After the surgery, all cases were prescribed steroid–antibiotic drops administered every four hours for 14 days, with a gradual reduction over the subsequent 6–8 weeks. In addition, cycloplegic eye drops were applied one or two times per day for 2 weeks to minimize the postoperative inflammation in the initial recovery phase after the surgery. Patients were followed up on the first day postoperatively, 1 week postoperatively, and 1 month postoperatively.

### 2.2. Statistical Analysis

Statistical analysis was performed, making use of SPSS Version 26 (IBM Inc., Chicago, Illinois, USA). Quantitative parametric data were presented as mean and standard deviation (SD) and were analyzed using the ANOVA (F) test with post hoc test (Tukey). In addition, quantitative nonparametric data were presented as median and interquartile range (IQR). They were analyzed using the Kruskal–Wallis and Mann–Whitney tests to compare each group. Qualitative variables were presented as frequency and percentage (%) and analyzed using the chi‐square test. Moreover, the correlation between various variables was done using the Pearson moment correlation equation for the linear relation of normally distributed variables. A two‐tailed *p* value < 0.05 was considered statistically significant.

## 3. Results

The study was conducted from January 2024 to June 2024 on forty eyes of forty patients who were aphakic without capsular support, either traumatic or due to complicated cataract surgery. Patients were divided randomly into two groups. Group A had the Yamane technique, and Group B had the prolene mesh technique. All patients were above 40 years old.

Patients in both groups were comparable in age, with a mean of 55.78 (± 8.63) for Group A and 59.10 (± 9.95) for Group B, and gender. In Group A, 50% were males and 50% were females, while in Group B, 60% were males and 40% were females. Systemic comorbidities that did not interfere with ocular health or visual potential such as well controlled diabetes mellitus without retinopathy, controlled hypertension, stable cardiovascular disease, and hyperlipidemia were not considered exclusion criteria and were recorded as baseline characteristics in Table 1, and none of them showed statistically significant difference between both studied groups.

**TABLE 1:** Patient demographic data.

		**Group A (*n* = 20)**	**Group B (*n* = 20)**	**p** **value**

Age		55.78 (±8.63)	59.10 (±9.95)	0.446

Gender	Male	10 (50%)	12 (60%)	0.530
	Female	10 (50%)	9 (40%)	0.754

Systemic comorbidities	Diabetes mellitus (without retinopathy)	1 (5%)	2 (10%)	1.000
	Controlled hypertension	4 (20%)	6 (30%)	0.716
	Hyperlipidemia	5 (25%)	5 (25%)	1.000
	Ischemic heart disease	1 (5%)	0 (0%)	1.000

*Note:* Pre‐UCVA: preoperative uncorrected visual acuity (log‐MAR), post‐UCVA: postoperative uncorrected corrected visual acuity (log‐MAR), Pre‐BCVA: preoperative best‐corrected visual acuity (log‐MAR), Post‐BCVA: postoperative best‐corrected visual acuity (log‐MAR), Pre‐IOP: preoperative intraocular pressure (mmhg), Post‐IOP: postoperative intraocular pressure (mmhg), Pre‐cylinder: preoperative cylinder error (DC), Post‐cylinder: postoperative cylinder error (DC), Pre‐sphere: preoperative spherical error (DS), Post‐sphere: postoperative spherical error (DS), IOL tilt using UBM (degree), complicated cases were excluded, Group A (Yamane group), Group B (Prolene mesh).

After the exclusion of complicated cases, both groups were compared for preoperative and postoperative (UCVA, BCVA, IOP, refractive errors, and IOL tilt) (Table 2). The two groups had no statistically significant differences (*p* value ≥ 0.05).

**TABLE 2:** Comparing pre‐ and postoperative values of both procedures.

	**Group A (*n* = 13)**	**Group B (*n* = 16)**	*p* **value**

Pre‐UCVA	0.02 (±0.01)	0.02 (±0.01)	0.654
Post‐UCVA	0.30 (±0.09)	0.28 (±0.10)	0.633
Pre‐BCVA	0.26 (±0.11)	0.23 (±0.05)	0.544
Post‐BCVA	0.45 (±0.13)	0.43 (±0.09)	0.690
Pre‐IOP	20 (±3.08)	19 (±2.58)	0.457
Post‐IOP	20.11 (±1.83)	18.90 (±1.20)	0.115
Pre‐cylinder	2.47 (±0.79)	3.02 (±0.95)	0.184
Post‐cylinder	−0.17 (±2.59)	−0.48 (±2.96)	0.811
Pre‐sphere	12.00 (±1.73)	11.60 (±1.45)	0.595
Post‐sphere	−0.33 (±1.94)	−0.38 (±1.87)	0.963
IOL tilt	4.00 (±0.82)	4.42 (±0.80)	0.454

*Note:* *p* < 0.05 statistically significant, results expressed as mean ± standard error of mean (minimum‐maximum), or frequency (%), Group A (Yamane technique), Group B (Prolene mesh technique).

Complications encountered during the follow‐up period included IOL tilts more than 5° or decentration more than 0.2 mm detected via UBM and elevation of IOP. Seven patients out of twenty were complicated in Group A. Three patients had IOL tilt > 5°, two had IOL decentration > 0.2 mm, and two patients had elevated IOP (pseudophakic reverse pupillary block). Four patients out of twenty had complications in Group B. Two patients had IOL tilt > 5°, and the rest had IOL decentration > 0.2 mm (Figure 2).

FIGURE 2Final prolene mesh configuration with the intraocular lens (IOL) in place. Ultrasound biomicroscopy (UBM) images showing a centered IOL and a tilted IOL supported by the prolene mesh.

**Figure:**
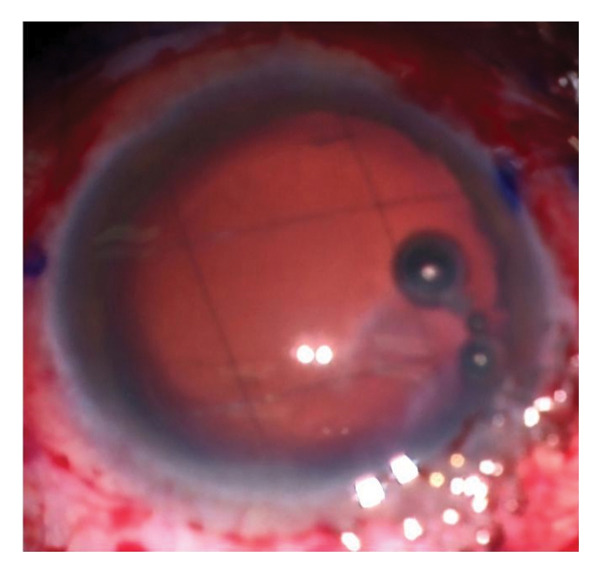
(a)

**Figure:**
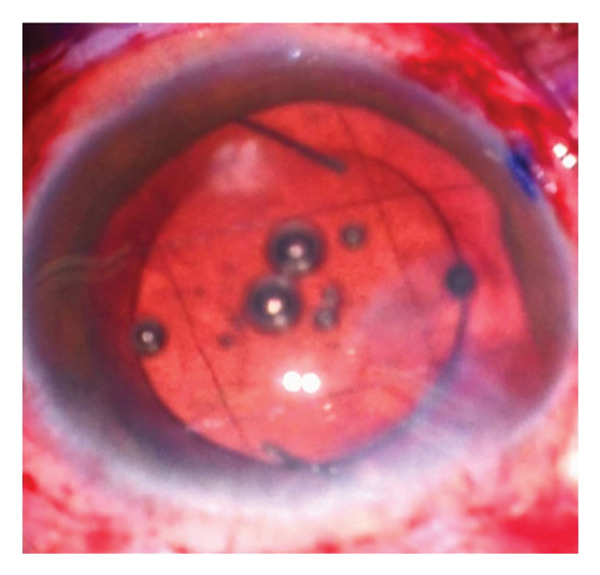
(b)

**Figure:**
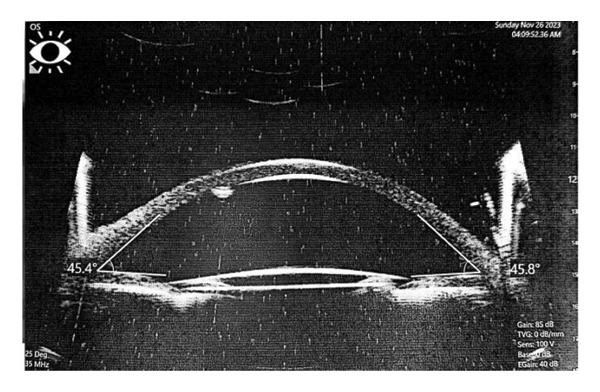
(c)

**Figure:**
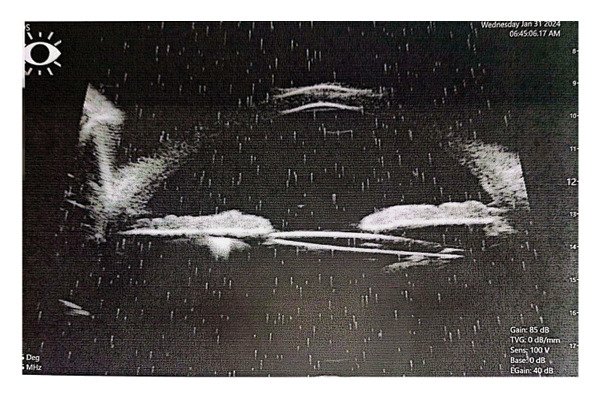
(d)

IOL decentration refers to the distance between the center of the IOL and the reference axis, whereas IOL tilt denotes the angle formed between the IOL axis and the reference axis. The reference axis is the corneal topographic center [10].

Refractive errors (sphere and cylinder) showed a strong negative correlation to IOL tilt of more than 5° for both groups (Tables 3, 4, and 5).

**TABLE 3:** Correlations between IOL tilt > 5° for Group A (Yamane technique) and postoperative refractive errors.

	***R***	*p* **value**

Post‐sphere	−0.99	0.02
Post‐cylinder	−0.99	0.02

*Note:* Post‐sphere: postoperative spherical error (DS), post‐Cylinder: postoperative cylinder error (DC), IOL tilt > 5° using UBM (degree), (*n* = 20).

**TABLE 4:** Correlations between IOL tilt > 5° for Group B (prolene mesh technique) and postoperative refractive errors.

	***R***	*p* **value**

Post‐sphere	−0.46	0.03
Post‐cylinder	−0.89	0.04

*Note:* Postoperative spherical error (DS), post‐cylinder: postoperative cylinder error (DC), IOL tilt > 5° using UBM (degree), (*n* = 20).

**TABLE 5:** Postoperative complications in both groups.

	**Group A (*n* = 7)**	**Group B (*n* = 4)**

IOL tilt > 5°	3 (15%)	2 (10%)
IOL decentration > 0.2 mm	2 (10%)	2 (10%)
Pseudophakic reverse pupillary block	2 (10%)	0

Figures 1(a) and 1(b) show the created 4 mm square 2^∗^2 prolene meshwork behind the pupillary plane before and after foldable three‐piece IOL implantation. Figures 1(c) and 1(d) show postoperative UBM in the prolene meshwork group with well‐centered IOL and IOL tilt.

## 4. Discussion

Secondary IOL implantation in the absence of capsular support is one of the challenges facing cataract surgeons. In such a situation, the challenge is to implant an unsupported IOL stably without tilting or decentration in a short time with the least possible complications.

Shin Yamane invented a technique for sutureless intrascleral IOL fixation by using needles to dock and externalize the haptics [11]. Many surgeons have adopted his technique, but they have encountered many complications that require preparation and management, such as choroidal hemorrhage, UGH syndrome, IOL decentration or tilt, lens capture, flagpole sign, and hypotony [12].

Some of these complications may be avoidable if we tried alternative techniques, adopting a concept of applying a rug that mimics complete capsular support. This idea was first described by Ayoub, who fashioned it at 1 mm posterior to the limbus, a transscleral DRSSR to support the IOL optic, providing a stable rug that can carry the IOL positioned at the sulcus [8].

In this study, we randomly divided enrolled subjects into two groups: Group A (Yamane technique) and Group B (prolene mesh technique). They were comparable in age and gender.

Both techniques improved uncorrected visual acuity, BCVA, and refraction (sphere and cylinder errors) without any statistically significant difference. They improved the final refractive status of the patients via secondary IOL implantation [13].

Both techniques showed within normal IOP postoperative measurements. Elevated IOP is a rare complication that may occur secondary to pseudophakic reverse pupillary block. The proposed mechanism involves a reduction in the space between the posterior surface of the iris and the IOL optic, leading to increased iridolenticular contact, development of reverse pupillary block, and subsequent elevation of IOP. In addition, chronic iris chafing against the IOL may result in pigment dispersion and anterior chamber inflammation, which can compromise trabecular meshwork function, impair aqueous outflow, and further contribute to increased IOP [14, 15].

In this study, IOP was within the normal range and comparable. We isolated those with elevated IOP and considered complicated cases of both groups to be compared to each other [16].

IOL tilt was comparable in both groups and within five degrees (5°), as we considered more than 5° tilt to be a complication and isolated to be compared in the complication table [17].

When we correlated the degree of IOL tilt to postoperative outcomes, Groups A and B showed strong negative correlations. Additionally, spherical and cylindrical errors were statistically significant.

Our findings were consistent with the literature. IOL tilt could affect the visual quality, the final refraction, and the amount of residual astigmatism [18].

Seven patients had complications in Group A, three had IOL tilt > 7°, two had decentered IOL > 0.2 mm, and two had pseudophakic reverse pupillary block. In Group B, two patients had tilted IOL > 7° and two had decentered IOL > 0.2 mm, while none had pseudophakic reverse pupillary block.

None of Group B showed elevation of IOP (pseudophakic reverse pupillary block), which could be explained by the privilege offered by the prolene mesh as the IOL lies posteriorly on the sulcus away from the pupil and the back of the iris [19].

## 5. Conclusion

Both techniques achieved comparable improvements in postoperative refractive error and final visual acuity. However, IOL tilt and decentration were more frequent with the Yamane technique, including a 10% rate of pseudophakic reverse IOL, whereas no such cases occurred in the prolene mesh group. Overall, the prolene mesh technique provided visual outcomes similar to Yamane but with fewer IOL stability–related complications, suggesting it may offer a safer and more predictable alternative for eyes without capsular support.

## Author Contributions

Islam Awny and Alaa Abdalsadek Ahmed Sinjab conceived and designed the study, performed the surgical procedures, and supervised the overall research process. Islam Saad EL Saman contributed to patient recruitment, data collection, and postoperative follow‐up. Elshimaa A. Mateen Mossa was responsible for data analysis, statistical interpretation, and preparation of the Results section. Amr Mounir participated in manuscript drafting, literature review, and critical revision of the manuscript for important intellectual content. All authors agree to be accountable for all aspects of the work.

## Funding

The researchers did not receive any private or public funding.

## Disclosure

All authors have read and approved the final manuscript. This paper has not been presented in a meeting. We clarify that a novel scientific content is presented in the paper, which differs totally from our previous publications in the journal about the same issue.

## Ethics Statement

In this manuscript, all ethical and administrative declarations have been clarified. Written informed consent for publication of anonymized clinical data was obtained from all participants.

## Conflicts of Interest

The authors declare no conflicts of interest.

## Supporting Information

Supporting Video 1 demonstrates the step‐by‐step surgical technique of the transscleral prolene mesh for secondary intraocular lens fixation in aphakic eyes lacking capsular support. The video illustrates the creation of the rectangular prolene meshwork posterior to the pupillary plane, passage of the sutures through partial‐thickness sclera, knot burial, and final placement of the three‐piece intraocular lens over the mesh to achieve stable optic support and centration (Supporting Video 1).

## Supporting information


**Supporting Information** Additional supporting information can be found online in the Supporting Information section.

## Data Availability

The data that support the findings of this study are available from the corresponding author upon reasonable request.
